# Supplementary material for: Regulation of Grain Chalkiness and Starch Metabolism by FLO2 Interaction Factor 3, a bHLH Transcription Factor in Oryza sativa
Source: Int J Mol Sci. 2023 Aug 14;24(16):12778. doi: 10.3390/ijms241612778 (PMC10454616; doi:10.3390/ijms241612778)
Supplement: Supplementary file 1 [file ijms-24-12778-s001.zip › ijms-2510738-supplementary.pdf]

International Journal of
Molecular Sciences

MDPI

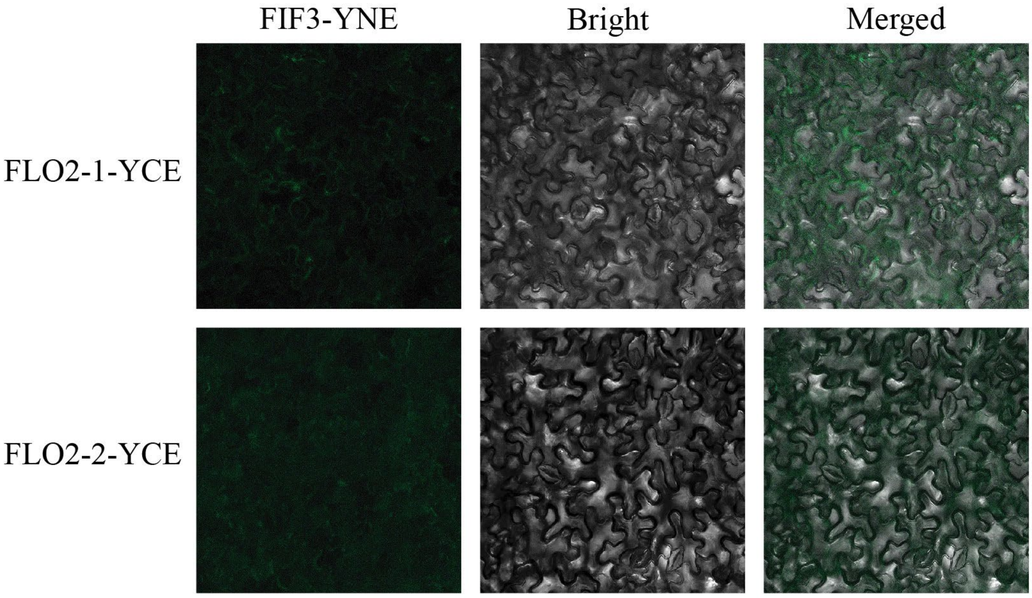

Supplementary Figure S1. BiFC result of *Os*FIF3 with FLO2. *Os*FIF3 can interact with longer FLO2-1 variant, but it is difficult to interact with shorter FLO2-2 variant.

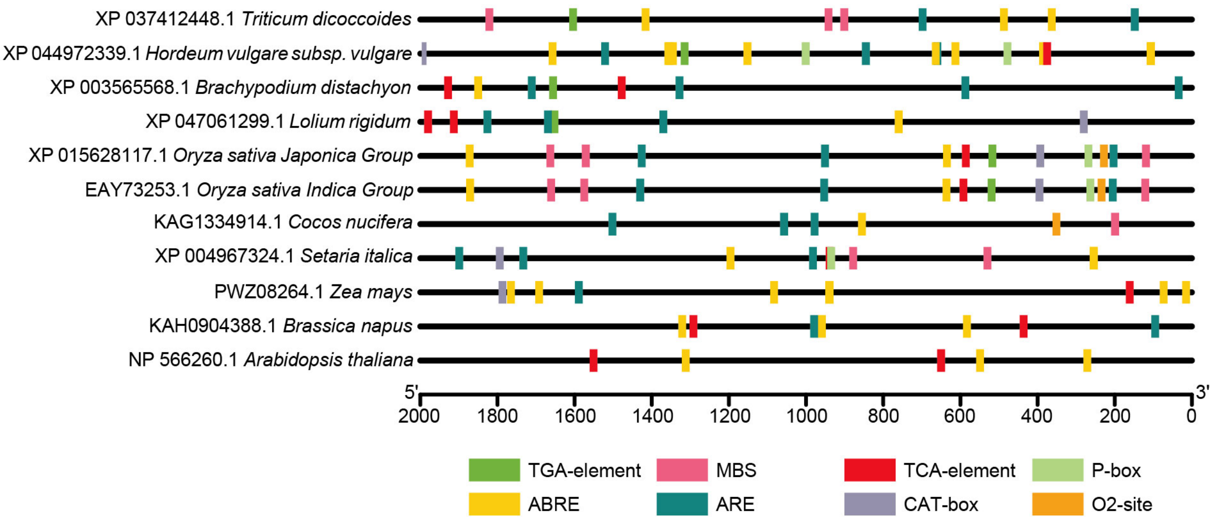

Supplementary Figure S2. Promoter alignment of *Os*FIF3 homologues from *O. sativa Indica*, *B. distachyon*, *T. dicoccoides*, *H. vulgare* subsp. *vulgare*, *L. rigidum*, *S. italica*, *C. nucifera*, *Z. mays*, *B. napus* and *A. thaliana*.

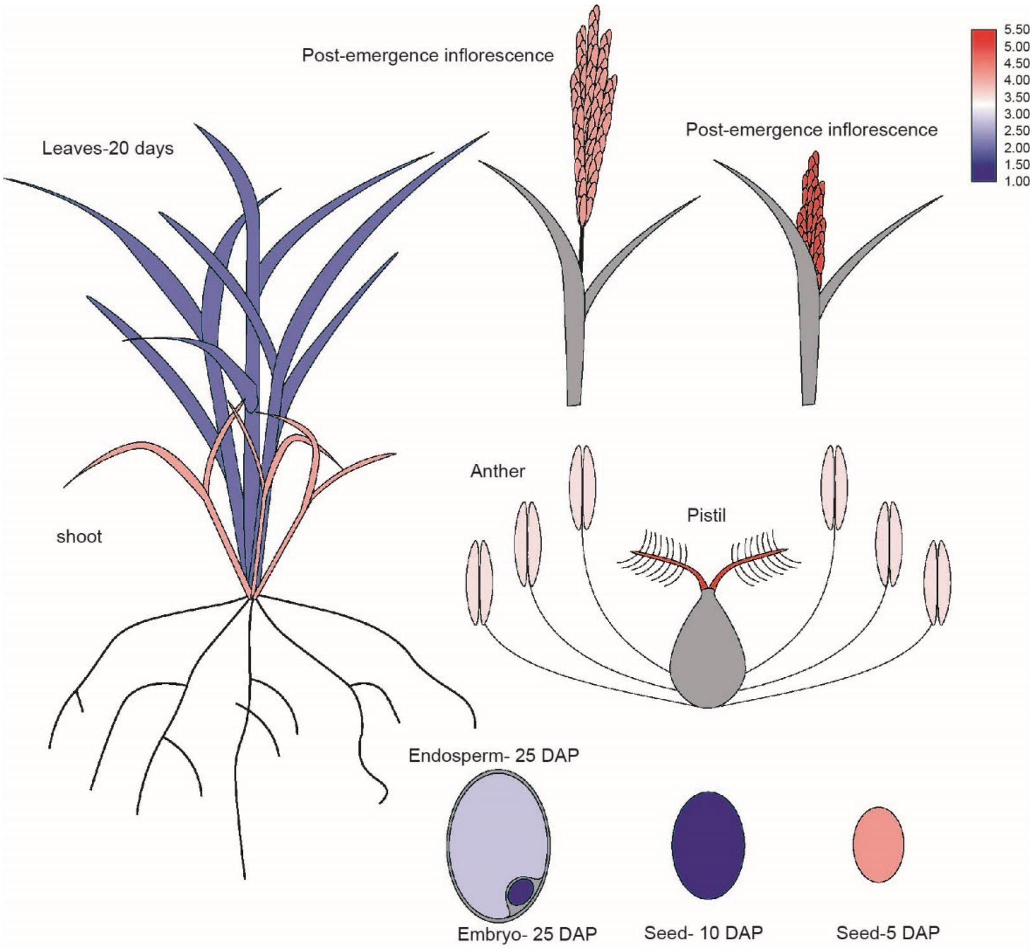

Supplementary Figure S3. Expression Patterns of *OsFIF3*. The eFP browser displays the expression pattern of *OsFIF3*, including shoots, leaves-20 days, post-emergence inflorescence, pre-emergence inflorescence, anther, pistil, seed-5 DAP, seed- 10 DAP, endosperm- 25 DAP, embryo-25 DAP. The expression level is quantified by Log2. *OsFIF3* expression is concentrated in shoots, post-emergence inflorescence, pre-emergence inflorescence, pistil and seed-5 DAP. This data is sourced from the Rice Genome Annotation Project, which can be found below this page (http://rice.uga.edu/cgi-bin/ORF_infopage.cgi?orf=LOC_Os01g14110). Expression level is RNA-Seq FPKM [12].

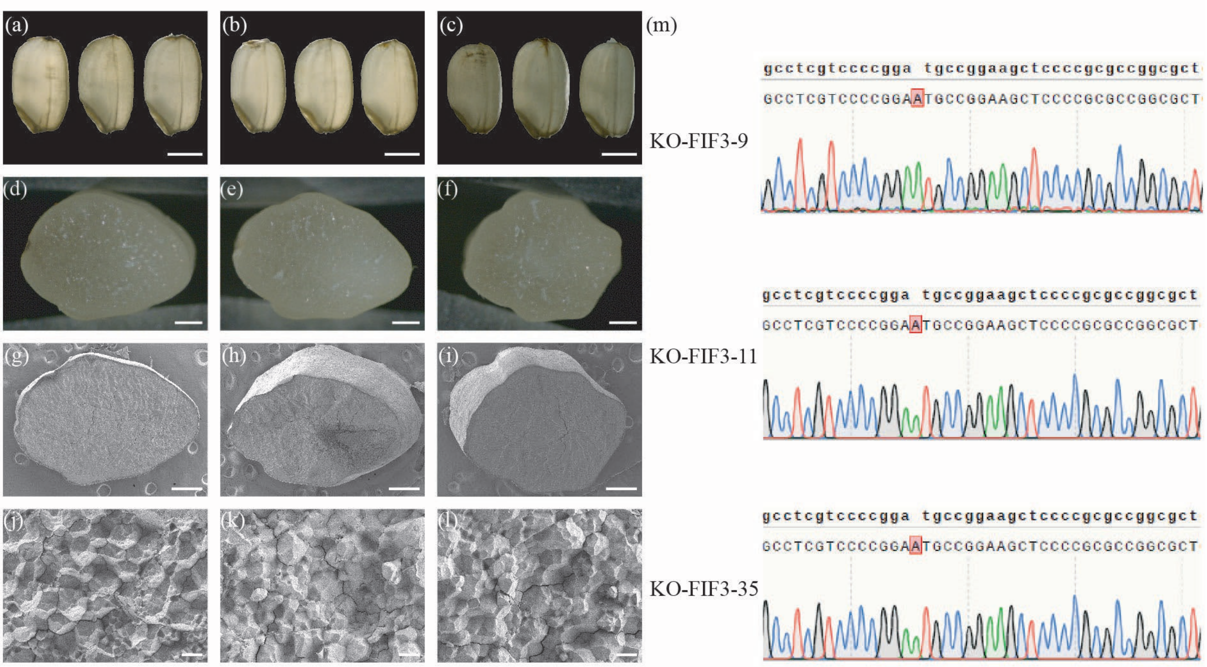

Supplementary Figure S4. Grain phenotype of *OsFIF3* knockout mutants. Grains of (a) *KO-FIF3-9*, (b) *KO-FIF3-11*, (c) *KO-FIF3-35* mutant under backlight conditions. Bar = 2 mm. Sectioned grains of (d) *KO-FIF3-9*, (e) *KO-FIF3-11*, (f) *KO-FIF3-35* mutant. Bar = 500 µm. Cross section of (g, j) *KO-FIF3-9*, (h, k) *KO-FIF3-11*, (i, l) *KO-FIF3-35* mutant grains under scanning electron microscopy. (g, h, i) Bar = 500 µm and (j, k, l) Bar = 15 µm. (m) Editing site of *KO-FIF3-9*, *KO-FIF3-11*, *KO-FIF3-35* sequence. One nucleartide (A) was inserted at the 522 of *OsFIF3*, causing frameshift in *fif3* mutants.

(a)

(b)

(c)

(d)

(e)

(f)

(g)

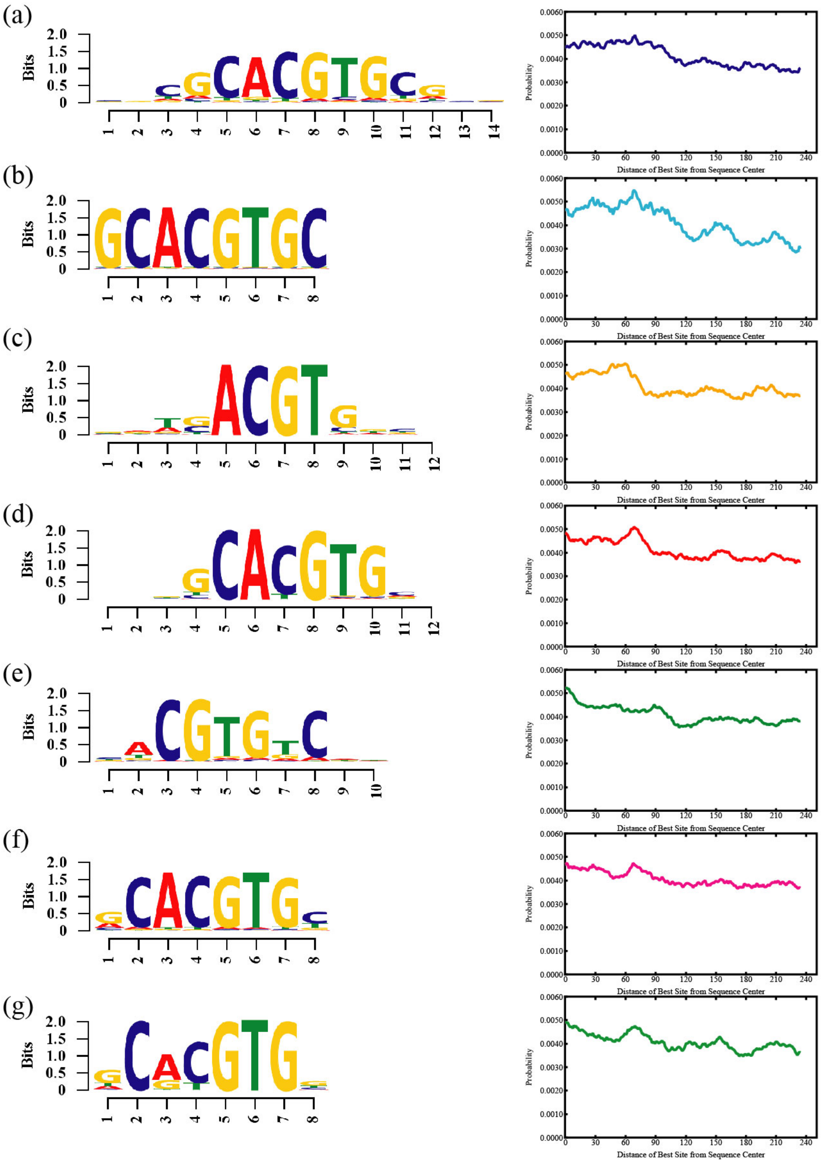

Supplementary Figure S5. CentriMo results of filtered DAP-Seq data (Site fold enrichment>13). Motifs of (a) bHLH145, (b) bHLH78, (c) HYH, (d) HBI1, (e) ABF3, (f) bHLH3 and (g) bHLH34 that had the similar CACGTG binding motifs were enriched in binding site.

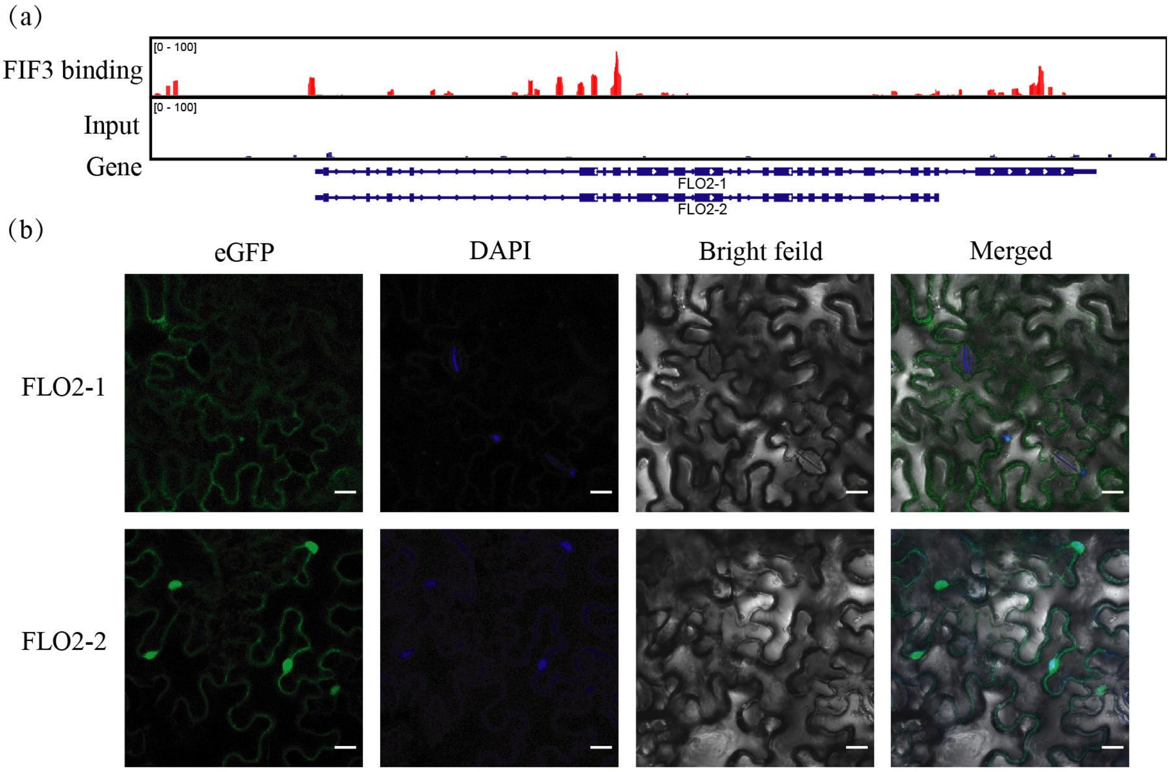

Supplementary Figure S6. DAP-seq of *Os*FIF3 and subcellular localization of *Os*FLO2. (a) A certain enrichment of *Os*FIF3 at the last exon of *FLO2* in DAP- seq. The structure of the exon was different between the two transcripts of *FLO2*. *FLO2-1* is a transcript variant containing the last exon in *FLO2*, whereas *FLO2-2* losses the last exon compared with *FLO2-1*. (b) The subcellular localization of the two FLO2 variants. The shorter variant FLO2-2 had more nuclear localization signals. Bar = 10 μm.

Supplementary Table S1. Statistical table of Dap-seq original data and quality control data.

	**Input_original**	**Input_ clean**	***Os*FIF3_original**	***Os*FIF3_clean**
Total Reads Count	24757336	22348416	21059742	20158164
Total Bases Count(bp)	3713600400	3324338832	3158961300	2957654918
Average Read Length (bp)	150	149	150	147
Q20 Bases Count (bp)	3629094088	3242148675	3075948728	2877564327
Q20 Bases Ratio (%)	97.72%	97.53%	97.37%	97.29%
Q30 Bases Count (bp)	3463524173	3084429315	2917864991	2726350220
Q30 Bases Ratio (%)	93.27%	92.78%	92.37%	92.18%
GC content (%)	50.36%	50.30%	54.86%	54.87%

Supplementary S1. GO enrichment standard.

Supplementary S2. KEGG enrichment standard.

Supplementary S3. Bed file of FIF3 binding sites. Headers for each column are chromosome, start, end, name and fold enrichment, respectively.

Supplementary S4. CentriMo result of FIF3 binding sites.
